# Developing Eradication Investment Cases for Onchocerciasis, Lymphatic Filariasis, and Human African Trypanosomiasis: Rationale and Main Challenges

**DOI:** 10.1371/journal.pntd.0002446

**Published:** 2013-11-07

**Authors:** Fabrizio Tediosi, Peter Steinmann, Don de Savigny, Marcel Tanner

**Affiliations:** 1 Swiss Tropical and Public Health Institute, Basel, Switzerland; 2 University of Basel, Basel, Switzerland; Imperial College London, Faculty of Medicine, School of Public Health, United Kingdom

## Background

The global health community pays renewed attention to evaluating the feasibility of elimination and eradication of certain communicable diseases [1], [2] besides continuing to reduce the burden of ill-health. Eradication depends on both the availability of tools to interrupt transmission, the capacity of health systems to implement these solutions effectively across all populations concerned, the required resources, and sustained political will. While the health and economic benefits of disease elimination and subsequent eradication may be substantial, elimination initiatives represent resource-intensive efforts with associated opportunity costs [3], [4]. Given the increasingly intense competition for global health resources, the decision to commit to national/regional elimination or eventual global eradication initiatives needs to be based upon robust analysis of benefits, risks, and costs.

Following an initial proposal of the Ernst Strüngmann Forum, convened in 2010 to explore the prospects, feasibility, and challenges of disease eradication [2], a working group developed a Guide to Preparing an Eradication Investment Case (EIC) [5]. The Guide proposes a generic approach applicable to any potentially eradicable disease.

Among the diseases tentatively identified as amenable to eradication are several neglected tropical diseases (NTDs) [6]. The vision of eliminating and eradicating selected NTDs has gathered momentum over recent years. In 2011, the WHO Strategic and Technical Advisory Group for Neglected Tropical Diseases and its partners adopted a roadmap for the control, elimination, and eradication of many NTDs. The global financial support for NTDs control and elimination is still comparatively low but has recently started to substantially increase. Following major pledges by the US Agency for International Development (USAID) (350 million USD for the period 2009–2013) and the UK's Department for International Development (DfID) for 2011–2015 (245 million GBP), the Bill and Melinda Gates Foundation (BMFG) donated 363 million USD for NTDs control and elimination in January 2012 (London Declaration, http://endtheneglect.org/2012/01/ntds-take-spotlight-at-london-declaration-meeting/).

## Eradication Investment Cases: An Innovative Method to Assess Global Health Investments

The essence of economics, namely to study how societies make resource allocation decisions, is answering three fundamental questions: 1) What products and services to produce? 2) How to produce them—adopting which production processes? 3) For Whom to produce products and services (who should benefit from the production and use of these products and services)? The first and second questions are related to the concept of efficiency—allocative and technical—while the third also addresses equity and fairness issues. The overall objective is usually maximizing social benefits, taking into account distributional effects or equity, with different societies placing different weights to efficiency and equity objectives.

An EIC is essentially an economic composite assessment in the broader meaning of economics, addressing thus all three fundamental economics questions. An EIC in fact answers: 1) the "What" question, that compares remaining in control mode versus moving toward elimination and then eradication; 2) the "How" question," assessing which intervention/s or strategy/ies should be adopted by which stakeholder, how much resources would be required, and how they could be mobilized (priority setting and resources allocation, funding); and 3) the "for Whom" question, assessing who would benefit from control or elimination in terms of health and economic gains, and the likely impact on equity and fairness.

An EIC is an approach to assess global health investments that, following the Guide [5] mentioned above, is structured into three main components:

1. **A description of the proposed investment**, providing a summary of the specific problem that the EIC is addressing and describing scenarios which, if properly implemented, could lead to elimination and ultimately eradication of the disease.
2. **The rationale for investing**, documenting the evidence on the biological, technical, social, and political feasibility, the estimates of the costs of potential approaches and their health and economic impact, the demand for elimination/eradication, and willingness to cooperate at the global, national, and local levels.
3. **The management and governance** aspects of the elimination/eradication initiative, describing the managing agency, the organizational arrangements, the role of all relevant partners involved in the elimination/eradication initiative at all levels—global, national, and local—including their responsibilities—e.g., technical support, monitoring and evaluation, etc.—and an assessment of their capacity.

This article presents the rationale, the approaches to be pursued, and the main methodological challenges of developing EICs for three neglected tropical diseases considered as candidates for elimination and eradication [4]: onchocerciasis (river blindness), lymphatic filariasis (LF) (elephantiasis), and human African trypanosomiasis (HAT) (sleeping sickness). Clearly, an EIC is a broad and innovative methodology to assess the potential consequences of investing in elimination and eradication of a disease. It is aimed at going beyond the traditional reductionist approaches focusing on only one or few dimensions—e.g., the health impact or the cost-effectiveness—that are relevant for informing policy decisions. Nevertheless, developing EICs, following the approach proposed by the Guide [5] mentioned above, presents several challenges.

## Challenges to Developing EICs for Onchocerciasis, LF, and HAT

There are several methodological challenges to developing EICs in general, and for onchocerciasis, LF, and HAT in particular. These challenges can be grouped into nine broad categories that are outlined below.

### Developing Realistic Scenarios to Move from Control to Elimination and Eradication

Developing EICs requires that scenarios be defined which can then be compared. A scenario is a full description of all activities required to achieve the intended outcome (i.e., control, elimination, or eradication) of the target disease if comprehensively and diligently implemented for as long as might be required to reach the desired outcome, sometimes decades later. Scenarios outline different options to move from control to elimination using different mixes of integrated interventions tailored to a given endemic setting.

In the EICs, the counterfactual scenario is always the current strategy to combat a given disease, implemented at the current intensity and coverage. Three broad scenarios are then comparatively assessed:

- Maintaining the current control (or elimination) efforts;
- Improving effectiveness and scaling up current control (or elimination) strategies to faster achieve elimination in given settings by using different strategies/intervention mixes;
- Progressive extension of the disease-free area until eradication is achieved using proven effective intervention mixes but tailored to the setting.

The scenario characterization includes a list of country-specific parameters describing the epidemiological situation and past efforts to combat the disease, operational thresholds defining the transition between program stages (e.g., from control to elimination mode, from periodic treatment to surveillance and response mode, etc.), pre- and post-elimination activities such as surveys to determine the epidemiological situation throughout the elimination phase and to attain and sustain elimination, and the anticipated need for routine health system–based disease surveillance to rapidly detect—and respond to—an eventual reemergence of the disease.

The scenarios for moving from control to elimination and eradication of onchocerciasis, LF, and HAT are built on a comprehensive and systematic historical review of the evidence on the local epidemiology, achieved population and regional coverage of interventions to control the diseases, and the achievements of the major global initiatives set up to control them. The review collects and analyzes data available from the WHO NTDs programs and from any possible disease-specific global initiatives. It includes data by country and region, mapping the areas of overlap of onchocerciasis, LF, and HAT, as well as other NTDs and additional major infectious diseases, and assessing the implications of ongoing control strategies and their escalation to elimination and eradication strategies.

The feasibility analysis of eradicating onchocerciasis, LF, and HAT considers the biological, epidemiological, social, economic, and political aspects in line with the criteria defined by the International Task Force on Disease Eradication and the consensus found by the Ernst Strüngmann Forum [7]. The biological and epidemiological criteria include epidemiological vulnerability, availability of effective practical interventions likely to achieve eradication, and demonstrated feasibility. The social and political criteria include a broad appreciation of the social importance of the disease, reasonable projected costs, synergies with other health system activities, and the contextual and political urgency for elimination/eradication rather than control.

The definition of scenarios and the analysis of the feasibility of elimination and eradication require simplifications of real-life conditions—i.e., a strong and coherent focus on the minimal essential information of the epidemiological, operational (implementation), and contextual conditions. The scenarios depend largely on existing data that often are not of the desired quality and not available for all settings. Predictions over years or even decades must then be made based on the available data, magnifying any imperfection in baseline data and requiring robust sensitivity analysis. This pragmatic approach leads to the best possible equilibrium between precision and feasibility. Moreover, the scenarios compared are developed in consultation with all interested stakeholders to reach consensus and wide acceptance among the global public health community and its major stakeholders which in turn renders an EIC widely credible.

### Global Analyses of Long-Term Impacts

The EICs are global analyses that in principle would require data from all endemic countries. However, these data are often only partially available and/or not of the quality needed. Consequently, respective adjustments and generalizations have to be made. Similarly, operational thresholds defining the transition between program activities need to be defined, but it must be considered that they do not always correspond to local epidemiological thresholds, e.g., transmission breakpoints may vary while the thresholds allowing cessation of periodic treatment and commencement of surveillance need to be standardized for operational reasons. Disease control approaches ideally protect individuals and reduce public health burden. They may as well have some partial effects on transmission. Elimination and eradication instead have an immediate impact on the transmission and thus also lower the burden, but moreover also protect future generations from the risk of infection, making future control measures unnecessary. The impact of disease control can therefore be measured in a shorter time frame, while the impact of elimination and eradication should be primarily evaluated based on its long-term effects.

Consequently, EICs require long-term predictions and imply decisions on how much value to give to future generations' health benefits and costs. While acknowledging the rather controversial aspect of whether or not to discount future health benefits and costs, and if yes, whether to discount benefits less than costs, the long-term dimension also—and equally importantly—requires assessing long-term political feasibility of the initiatives which includes the long-term reactions of all stakeholders involved. Therefore and at a practical level, EICs must adopt robust methods and approaches to assess the impact of uncertainty in models and parameters used.

### Disease Elimination and Eradication Are Global Public Goods

Disease elimination and eradication are global public goods. It is not, in fact, possible to exclude a country or a community from the benefits of eradication efforts, and every country/community can benefit from eradication efforts without limiting the others' benefits. As a consequence, each country's decision to eliminate a disease is likely to depend on whether other countries also are eliminating/have eliminated the disease. In order to achieve eradication, all countries need to engage in elimination. If only one country does not do it, then eradication cannot be achieved. Thus, coordination and incentives to coordinate are of paramount importance. Interdependence of decisions to eliminate mainly depends upon geographical circumstances. Clearly, for countries that share borders the success of elimination depends also on the decision of the neighbors to eliminate. As the majority of NTD-endemic countries are neighbors, the decisions to control or eliminate are often deeply interrelated. In EICs therefore, coordination and incentives to coordinate are important factors to be taken into account as only with an effective cooperation at global, national, and local levels is it possible to move from control to elimination. Hence, the EICs will assess for each disease how critical cooperation is for success at the various levels, particularly with regard to sharing information about surveillance, diagnostics, forecasting of demand of commodities for elimination, and coordinating purchases. The analysis will focus specifically on: (i) the potential role of all various international agencies in the global effort to eliminate and then eradicate onchocerciasis, LF, and HAT; (ii) the nature and extent of the required commitment of government agencies; (iii) the political support required at local levels; and (iv) the available mechanisms for structuring robust, durable agreements.

### Assessing Long-Term Health Impacts

The long-term health impact of the scenarios can be assessed using epidemiological models that include estimates for transmission interruption thresholds and consider the risk of reemergence of the disease as well as the potential implications of rising life expectancies over time. A recent review article showed that more research is needed to develop methods to link dynamical models of infection and disease, parameterize models to allow greater location specificity, and better understand long-term effects of drug efficacy on parasite populations and morbidity, allowing in turn robust health impact assessments and cost-effectiveness analyses [8].

For onchocerciasis, the assessments and predictions developed so far involved stochastic microsimulations to calculate the life events of individual persons and colonizing parasites, or deterministic vector models of the dynamics of the *Simulium* populations and the development of the parasite in the black flies [9]–[11]. Based on field observations and the results of the carefully parameterized model ONCHOSIM, elimination of onchocerciasis from most endemic foci in Africa appears to be possible. However, the same model suggests that the requirements for global eradication in terms of intervention duration, coverage, and frequency of treatment may be prohibitive given the current tools and under current funding levels, particularly in highly endemic areas [12].

Similar modeling approaches have been applied to LF [13]–[15]. Particular consideration needs to be given to the existence of the three different LF species (*Wuchereria bancrofti*, *Brugia malayi*, and *B. timori*), endemic in different places and transmitted by different vectors. There have been several modeling exercises addressing the dynamics of LF transmission in specific localities [16].

A deterministic model of HAT suggests that the vector numbers generally need to be reduced by 90% to achieve local elimination [17]. An agent-based model has been used in order to incorporate the spatial dimension of transmission [18], but ecological differences between endemic foci obviously affect the feasibility of tsetse control. These have received little consideration in dynamic models of HAT. There also remain important uncertainties about disease progression which may have substantial implications [19], [20]. There are also important differences between *T.b. gambiense* and *T.b. rhodesiense* in terms of the significance of the animal reservoir, human infectiousness, and pathology [20]–[22], all of which need to be taken into consideration.

Finally, the health impact of the different scenarios can be measured in terms of number of cases, deaths, and burden averted (expressed in DALYs) which in turn allow comparisons with the impact of interventions to control other diseases [23].

### Assessment of Financial and Economic Costs

Another set of challenges are related to estimating the financial and economic costs of the interventions to eliminate and eradicate a disease. The total financial and economic costs of the various scenarios include capital and recurrent costs for all core categories. Further disaggregation by country income group (e.g., using World Bank income groups based on gross national income [GNI] per capita) may be used to illustrate asymmetries in costs (and benefits) as a function of country income. The costs will be presented stratified into those to achieve elimination and those to sustain elimination with the goal of eradication. Nevertheless, limited data are available on both financial and economic costs of interventions to control and eliminate onchocerciasis, LF, and HAT, and available data often do not distinguish between resource use and unit costs. Consequently, respective approximations with best and worst case estimates are made.

A further challenge is the limited evidence on how to take into account the potential for diminishing returns to scale and an increase in unit costs as programs are expanded to cover groups that are harder and harder to reach. Specific costing models should therefore be developed for estimating the costs of activities such as periodic surveys and surveillance, and to account for the needed initial investments.

The impact on labor productivity of these diseases is equally important and presents further challenges due to difficulties in both measuring and valuing this specific impact. For instance, troublesome itching and vision impairment due to onchocerciasis are associated with decreased productivity at individual level and a loss of labor and a degraded productivity at societal level. However, measuring the microeconomic impacts of ill health and generalizing them is prone to difficulties and controversies about the approaches to be adopted [24], particularly in the case of onchocerciasis, LF, and HAT that affect largely highly marginalized populations.

### Assessing Cost-Effectiveness and Broader Economic Impacts

Cost-effectiveness analyses yield estimates of the monetary costs of alternative means of producing an effect or outcome that needs not be measured in monetary terms [20], [25]–[27], for example the cost of immunizing a child or the cost per healthy year of life gained. In the EICs, the cost-effectiveness analysis takes a societal perspective, capturing the costs and benefits before and after achieving elimination. The framework for the benefit-costs assessment thus extends the net-benefit framework to include an additional term, the net benefits of interrupting transmission to achieve elimination.

Both costs and effects occurring in the future are discounted using different discount rates and the implications of chosen discount rates on the results are assessed. It has been suggested that constant discounting may undervalue the future [5]. Given the relevance of discounting in the EICs, the impact of applying a discount rate for health effects that is lower than that of costs, but above zero, should be tested. In addition, the results should be presented after applying a nonconstant discount rate (declining or "slow" as opposed to exponential discounting; i.e., discounting at a constant rate) [5], allowing decision makers to appreciate the impact of these methodological choices on the results.

The sensitivity of the economic analysis to possible variations in the values of critical parameters should also be examined. The parameters chosen for the sensitivity analysis relate to identified project constraints or critical risks and prediction errors (such as prices or discount rates). Probabilistic sensitivity analysis is run to account for parameter uncertainty.

Cost-benefit analysis requires monetary estimates of both costs and effects. Cost-benefit analysis is useful, for example where it is necessary to make intersectorial or cross-project comparisons such as a comparison between a health and education project. Nevertheless, how to extend cost-effectiveness analysis to cost-benefit analysis of health interventions is controversial. In particular, the use of the value of a statistical life to monetize benefits has been rejected by WHO and is therefore not adopted in our EICs. Instead, the EICs assess wider economic benefits of elimination/eradication. These include an assessment of the impact of interventions on economic productivity and development. The economic analysis explores the pathway(s) through which elimination or eradication can affect economic activity both at the individual household and population level, including the different mechanisms through which health can affect income (e.g., productivity, children's education, savings and investments, and demographic structure) [24].

The assessment of broader economic impacts of eliminating onchocerciasis, LF, and HAT presents further challenges. First, the impact at country level may not be very high because of the low and often very focal incidence and prevalence of these diseases. In addition, the available data and the methods for assessing the impacts on economic development are limited. For example, it is by definition not possible to carry out pre-post studies using quasi-experimental designs. Nevertheless, elimination of onchocerciasis, LF, and HAT are indeed expected to have an impact on economic growth and development through the effects, for instance, on the agricultural sector and on human capital accumulation. However, it is not only hard to find good indicators that capture these impacts, but also current control efforts have already brought substantial economic benefits and so further benefits of elimination/eradication might be smaller than expected.

### Assessing Broader Social Impacts

The eradication of onchocerciasis, LF, and HAT might have positive and negative social impacts. The social impact includes intergenerational benefits and the contribution of elimination/eradication of these diseases to attain public goods such as reducing global health inequity.

Positive social impacts may include the elimination of stigma; economic and quality of life improvements; increases in mobility and productivity, in education attainments, and in access to care; and structural improvement of health care services, community participation, and democracy. A negative impact may result from how the project is implemented, e.g., from coercion, fear, an excessive pressure/workload of health personnel, and the diversion of funds from more beneficial areas of intervention.

A recent systematic review conducted by the Swiss TPH documented social impacts of onchocerciasis, LF, and HAT [28]. It analyzed the evidence on: a) the psychosocial and social impacts of the diseases; b) the social determinants of these diseases at community level, including social inequalities; c) people's knowledge, practices, and health-seeking behavior related to the three diseases; and d) social aspects related to the programs, particularly the social and micropolitical contexts of community-directed drug distribution (in onchocerciasis and LFs programs) and its influence on the development and functioning of the program, and the social factors that enable or hinder coverage and adherence to medication. The review revealed that these impacts, although important, are however hard to measure and even harder to quantify.

### Modeling Interactions between Disease Control Programs

Another set of challenges for developing these EICs for onchocerciasis, LF, and HAT are related to the fact that the elimination and eradication initiatives will be happening in a context of, sometimes rapid, scale up of different interventions to control or eliminate other NTDs as well as other infectious diseases such as malaria. In many areas, onchocerciasis and LF are co-endemic, and scaling up coverage of interventions to prevent or treat one of them will result in substantial impact on the other as the same drug, i.e., ivermectin, is used for the treatment of both. This is also true for other diseases such as malaria where the ongoing initiatives to scale up vector control may have substantial impacts on LF [29]. Thus, the integration of control and elimination initiatives must be taken into account and the impact evaluated not only on the epidemiology of the diseases but also from a programmatic point of view, e.g., considering local capacity and health systems. In addition, economic consequences of program integration must be carefully evaluated.

### Assessment of Short- and Long-Term Health Systems' Needs and Impacts

Health systems play a crucial role in achieving control, elimination, and then eradication of onchocerciasis, LF, and HAT, and hence their capacity in large part determines the feasibility of reaching these goals. The historical experience of onchocerciasis control shows how difficult it is to sustain high treatment coverage with ivermectin, even when the drug is donated. Communities play a decisive role in the community-directed treatment (CDTi) strategy, implying that health systems must empower local communities as well as health services. CDTi is seen as an effective approach to strengthening peripheral and district health systems [30].

Interestingly, important differences exist between regions with regard to the most suitable avenues for mass drug administration. Similarly to onchocerciasis, LF control relies on the periodic administration of ivermectin (or Diethylcarbamazin (DEC)-mediated salt outside Africa) together with albendazole to a large fraction of the population residing in endemic areas. While CDTi appears to work reasonably well in many African settings, efforts to replicate this model in other regions mostly failed. Instead, in Asia, the Pacific, and the Western hemisphere distribution through the health care system is arguably more successful.

HAT is one of the few diseases where effective control depends on active screening to ensure the early detection of cases. Symptoms in the initial phase of the illness, when treatment has the greatest chance of success, are often mild or nonspecific. The capability of the health system to detect cases and respond quickly is thus very important. Eliminating HAT requires massive efforts in targeted active case detection and a substantial strengthening of passive case detection; the latter strengthens health systems so that misdiagnosis and underreporting are reduced. Finally, there must be a coordinated multisectoral approach to tsetse control that involves specialists in human and animal health, livestock, agriculture, tourism, wildlife, and vector control.

The concept of health systems effectiveness can be adopted to analyze health systems constraints to elimination [31]. The major health system factors can be grouped into those related to the availability of competent human resources; managerial capacity in general health services to plan, implement, and monitor interventions; procurement and supply chain management; delivery systems; disease surveillance and response; national and local information systems; involvement of communities in intervention delivery; and financing.

Integration of the elimination initiatives into national and local health systems, and thus into regular health planning, may be needed to scale up the interventions and sustain high coverage for long periods. There is also scope for integration of onchocerciasis and LF programs as both rely on similar delivery strategies and interventions and could therefore be managed and planned together.

Disease surveillance is increasingly important when the disease burden is lowered in the pre-elimination and elimination phases. Disease surveillance coupled with active response strategies can be seen as an intervention that needs to be effective and dynamic, and therefore integrated into functional health systems.

The health system impact assessment can be conducted following systems thinking principles [32]. Systems thinking helps to reveal the underlying characteristics and relationships of the key functions of the overall system. It is applied to anticipate how the control/elimination/eradication strategy might have system-wide effects across the systems building blocks—leadership and governance, health system financing, health workforce, health information systems, medical products, vaccines and technologies, and service delivery. It tries to identify synergies to strengthen the system and avoid detrimental unintended consequences that can be mitigated [33].

## Conclusion

The EIC framework provides a methodology by which the feasibility, costs, and consequences of elimination and eradication of candidate diseases can be assessed. The EICs aim to go beyond traditional efficacy and efficiency measures to take into account multiple dimensions of such endeavors, including their implications for health systems and wider economic benefits. Although promising, the EICs approach proposed by the recent Guide [5] has not yet been applied to any disease program. In this article, we described the approach and main challenges or boundary conditions to develop EICs for onchocerciasis, LF, and HAT. We show how the EIC approach goes beyond traditional efficacy and efficiency measures to take into account multiple dimensions. The results of the EICs for onchocerciasis, LF, and HAT will serve not only to inform decisions of global and national policy makers but also to test the applicability of the EICs framework at the national and global level.

**Building Scenarios for Eliminating and Eradicating Onchocerciasis, Lymphatic Filariasis, and Human African Trypanosomiasis**

**Onchocerciasis**

Onchocerciasis control relies on the control of the *Simulium* spp. vectors and the administration of ivermectin to at least 65% of the at-risk population for many years [34]. Recent evidence indicates that mass treatment with ivermectin is not only a strategy for controlling onchocerciasis as a public health problem, but that it can also interrupt transmission and eliminate the parasite in endemic foci if high treatment coverage can be maintained for a decade or more, depending on the local epidemiological situation [35]. For the purpose of the EIC, the scenario to move toward elimination of onchocerciasis is based on the current strategy of community-directed treatment with ivermectin (CDTi), with coverage extended to all areas where there is local transmission, i.e., a nodule prevalence >5% (traditionally, interventions focus on areas with a nodule prevalence >20%), and sustained mass treatment up to demonstrated elimination in the entire focus. Thereafter, periodic epidemiological and entomological surveys as well as passive surveillance need to be maintained pending global elimination (i.e., eradication). Maintaining mass treatment for the required duration is a major challenge in regions with weak governance and health system capacity.

**Lymphatic filariasis (LF)**

With regard to LF, spectacular results have been achieved in many settings where traditionally high prevalence and disease burden have been reduced through concerted efforts in vector control and mass drug administration, relying on ivermectin in Africa and DEC in other regions, both now usually co-administered with albendazole [36]. LF control targets all areas where local transmission of LF has been detected (e.g., though surveys). While LF control is well advanced in many countries in Asia, the Pacific, and in the Western hemisphere, implementation is much slower in Africa where a range of countries still need to update epidemiological maps and establish national programs targeting the entire at-risk population [37]. In order to be successful, elimination programs need to achieve coverage of the at-risk population in excess of 65%, and maintain it for several years, depending on the local epidemiological situation, vector fauna, and other factors (often around six years) [38]. Thereafter, regular surveys and surveillance are required in order to detect recrudescence.

The EIC scenarios take into account that in areas where onchocerciasis and LF are co-endemic (mainly in Africa), close collaboration is required between the two elimination programs as both programs rely on ivermectin distribution by community volunteers, require post-treatment surveillance, and drug distribution for either program cannot come to conclusion—and thus surveillance cannot be started—if the other program is continuing ivermectin treatment in the same area. Considering the generally much longer time horizon of onchocerciasis control programs compared to LF control programs, the duration of the former will be the decisive factor in most areas. On the other hand, it must be noted that LF has been found to reemerge after near-elimination in certain areas [39]. Last, LF elimination is furthered in areas where it is transmitted by *Anopheles* spp. mosquitoes by efforts to control malaria with long-lasting insecticidal-treated bednets (LLITNs). Other forms of vector control might also play a role even if not designed and implemented for LF control [40].

**Human African Trypanosomiasis (HAT)**

The scenarios for HAT control focus on eliminating the parasites causing human disease rather than the vector (tsetse flies). Hence, case detection (active and passive) and treatment are the mainstay of the scenarios, with these efforts supported by targeted interventions to reduce vector density with a view of reducing transmission (e.g., using insecticide-treated targets and cattle) [41]. Scenarios also take into account that while in *T.b. gambiense*–endemic areas eradication is conceptually feasible (none or very limited animal reservoir), in *T.b. rhodesiense*–endemic areas the mere concept of eradication is questionable as *Glossina* spp. (vector) elimination would need to be achieved due to the presence of extensive animal reservoirs, including in wild animals [42]. Thus, the scenarios for *T.b. gambiense* will aim at "eradication" while those for *T.b. rhodesiense* will aim at "elimination in humans." As a consequence of focusing on the parasite causing human disease and neither trypanosomes in general nor the tsetse flies transmitting them, the economic benefits of elimination and eradication will chiefly result from improved public health and reduced suffering rather than increases in livestock production and improved agricultural opportunities.
